# Characterization of exogenous DNA mobility in live cells through fluctuation correlation spectroscopy

**DOI:** 10.1038/srep13848

**Published:** 2015-09-10

**Authors:** Stephen Mieruszynski, Michelle A. Digman, Enrico Gratton, Mark R Jones

**Affiliations:** 1 University of Western Sydney, School of Science and Health, Hawkesbury Campus, Locked Bag 1797, Penrith NSW 2751, Australia; 2 Department of Developmental and Cell Biology, University of California Irvine, Irvine, California, United States of America; 3 Department of Biomedical Engineering, Laboratory for Fluorescence Dynamics, University of California Irvine, Irvine, California, United States of America; 4 Centre for Bioactive Discovery in Health and Ageing, School of Science and Technology, University of New England, Armidale, Australia.

## Abstract

The spatial-temporal dynamics of delivered DNA is a critical aspect influencing successful gene delivery. A comprehensive model of DNA lipoplex trafficking through live cells has yet to be demonstrated. Here the bioimaging approaches Raster Image Correlation Spectroscopy (RICS) and image-Means Square Displacement (iMSD) were applied to quantify DNA mechanical dynamics in live cells. DNA lipoplexes formed from DNA with a range of 21 bp to 5.5 kbp exhibited a similar range of motion within the cytoplasm of myoblast cells regardless of size. However, the rate of motion was dictated by the intracellular location, and DNA cluster size. This analysis demonstrated that the different transport mechanisms either had a size dependent mobility, including random diffusion, whereas other mechanisms were not influenced by the DNA size such as active transport. The transport mechanisms identified followed a spatial dependence comparable to viral trafficking of non-active transport mechanism upon cellular entry, active transport within the cytoplasm and further inactive transportation along the peri-nuclear region. This study provides the first real-time insight into the trafficking of DNA delivered through lipofection using image-based fluctuation correlation spectroscopy approaches. Thereby, gaining information with single particle sensitivity to develop a deeper understanding of DNA lipoplex delivery through the cell.

A comprehensive understanding of the spatial-temporal dynamics and molecular mechanisms behind DNA lipoplex dynamics in live cells is paramount for the further development of the gene delivery field. Mobility of delivered DNA and DNA lipoplexes is thought to be one of the major limitations in the delivery of foreign DNA sequences^1^. However, limited tools have been available to characterise DNA lipoplex mobility and dynamics from entry into and transit through the live cell, and into the nucleus with single particle sensitivity.

Although the process of lipoplex delivery has been well documented at specific locations or time points, the molecular dynamics of the delivered DNA has yet to be addressed throughout the entire cell. It is known that following entry into the cell, DNA lipoplexes are initially contained within endocytic vesicles and therefore, must escape these cellular organelles to achieve a gene therapy outcome^2^. Within the cytoplasm, the most efficient form of motion towards the nucleus is facilitated by motor proteins along the microtubule network^3^. If the DNA is unable to egress the endosomes and traffic to the nucleus, it is possibly degraded by nucleases^4^. Intact DNA must enter the nucleus, commonly described as the ultimate obstacle of gene delivery^5^,^6^, through nuclear pore complexes^7^, or by associating with chromatin during cell division^8^,^9^.

Recent advances in single cell confocal imaging make it possible to elucidate the molecular behaviour of fluorescently labelled particles based on their fluorescence fluctuations in both time and space^10^. Using fluorescence oscillations of individual particles, a number of techniques have been developed which include Raster Image Correlation Spectroscopy (RICS)^11^, image-Means Square Displacement (iMSD)^12^ and Number and Molecular Brightness (N&B)^13^. The RICS, iMSD and N&B are techniques based on the principles of Fluorescence Correlation Spectroscopy (FCS), which enable the quantification and extraction of information on the mobility^11^, mechanisms behind motion^12^ and particle number^13^ of fluorescently labelled particles, respectively.

The RICS approach works on the principle of applying a raster scan during acquisition. While acquiring images, the Point Spread Function (PSF) must overlap, and as a particle moves it will be observed in neighbouring pixels as the raster scans across. In the case of a slower particle, it is more likely to be observed in immediately adjacent pixels for a short period of time, resulting in a spatial correlation that is well resolved in adjacent pixels but decays as it is no longer observed. Whereas, a faster particle will be observed further in space, but is less probable to be observed in adjacent pixels, resulting in a characteristic spatial correlation that decays rapidly and broadens^11^. Through the application of the Spatial Autocorrelation Function (SCAF) approach, mobility coefficients are obtained through the fitting of data, thus enabling the quantification of particle mobility of an image series^11^,^14^.

The iMSD approach on the other hand, expands on the spatiotemporal image correlation spectroscopy (STICS) method in which the position of the average spatiotemporal correlation function is tracked over time with high spatial resolution (to the order of several tens of nanometers) providing an insight into the directed motion, flow and directionality of particles. However, iMSD differs significantly from the STICS approach as it fits data obtained from fast imaging, as the STICS approach can only elucidate the mobility of very slow moving particles and therefore, image series with a large time delay or slow sampling rates^12^. The MSD curves obtained from this analysis enable the differentiation between a number of mechanistic events, including random diffusion, active transport, anomalous subdiffusion, confined diffusion, transient confinement, and binding-unbinding^12^.

Previously, RICS, iMSD and N&B have been applied to study fluorescent protein dynamics in live cells^11^,^12^,^13^, and the N&B approach to quantify DNA lipoplex aggregation^15^. Here these methods have been applied to address and extract the spatial-temporal dynamics of lipoplex delivered DNA of various sizes in live cells. The RICS approach was applied to quantify the rate of motion, whereas the iMSD technique enabled the identification of how the DNA moves. The influence of the position of DNA, i.e. along the cell extremity, cytoplasm, peri-nuclear area and nucleus, was explored demonstrating these regions of the cell result in different rates of motion. Transport mechanisms were studied throughout the cell, showing a size dependent mobility in specific mechanisms and the spatial distribution of these mechanisms. The number component of the N&B approach was utilised to count the DNA particle number localised in the nucleus. This study presents a model to study DNA lipoplex dynamics through the entire cell, in real time and with single particle sensitivity.

## Results

### Applying RICS and iMSD to Extract Information on DNA Mobility

DNA of various lengths, 120, 240, 495, 1000 and 1985 bp; were produced through PCR using an Alexa Fluor488 (AF488) labelled primer, or 5.5 kbp DNA plasmids (linear and circular) were fluorescently labelled via an AF488 DNA labelling kit. These fluorescently labelled DNA (flDNA) constructs were used to form lipoplexes, and delivered to a myoblast progenitor stem cell line to study the spatial temporal dynamics of the delivered DNA. Muscle tissue presents as an ideal gene therapy target, not only for the treatment of muscular diseases, but to also act as factories to supply therapeutic factors systemically^16^.

**Figure 1:**
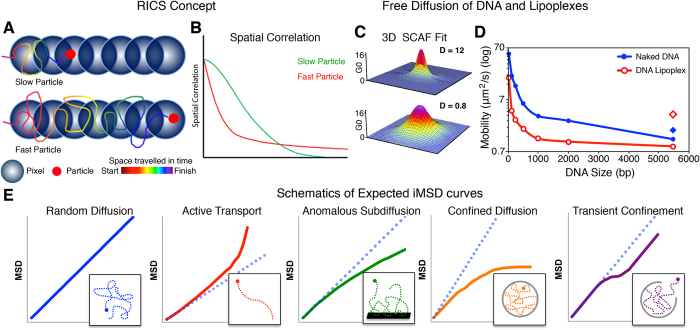
Characterisation of DNA Mobility with RICS and iMSD. (**A**) The RICS approach requires an image collected with a raster scan and works on the principle that a slower moving particle will be observed across fewer pixels compared to a faster particle resulting in a spatial correlation that decays earlier (**B**). (**C**) Mobility coefficients are obtained from the SCAF fit, as shown for the 240 bp fragment (top) and linear plasmid lipoplex (bottom) in solution exhibiting a diffusion of 12 μm^2^/s and 0.8 μm^2^/s, respectively. (**D**) Diffusion of naked DNA (blue, filled) and DNA lipoplexes (red, empty) in solution obtained from RICS analysis. In the graph presented, the diamonds represents the naked and lipoplex circular plasmid in blue and red, respectively. (n = 10 samples, and 10 observations for each). (**E**) Through the same image series collected for RICS, the iMSD approach can be applied enabling the mechanism of motion to be determined through the calculation of the MSD. Mechanisms of motion include random diffusion, active transport, anomalous subdiffusion, confined diffusion and transient confinement (left to right).

The free diffusion of the flDNA fragments was explored in solution as either naked DNA or as DNA lipoplexes. In the RICS approach, fitting of the data through the 3D SCAF provides quantification of fluorescently particles. Figure 1C presents the 3D SCAF obtained from the naked 240 bp fragment and 5.5 kbp linear plasmid lipoplex with a free diffusion coefficient of 12 μm^2^/s and 0.8 μm^2^/s, respectively. These two SCAF demonstrate the characteristic differences in RICS analysis output as mobility changes. In the faster 240 bp fragment a broadening in the SCAF *x* axis is observed, as opposed to the slower linear plasmid lipoplex, which exhibited better resolved SCAF in both the *x* and *y* axis.

Across the DNA sizes observed (21 bp to 5.5 kbp) a size dependent diffusion was demonstrated in solution through RICS analysis (Fig. 1D and Supplementary Fig. 1). The naked DNA fragments exhibited a more rapid diffusion rate compared to the DNA lipoplexes, which were on average 3-fold faster. However, the circular plasmid lipoplex showed a 2-fold faster diffusion than the naked circular plasmid.

### Quantification of DNA Dynamics within the Cytoplasm

**Figure 2:**
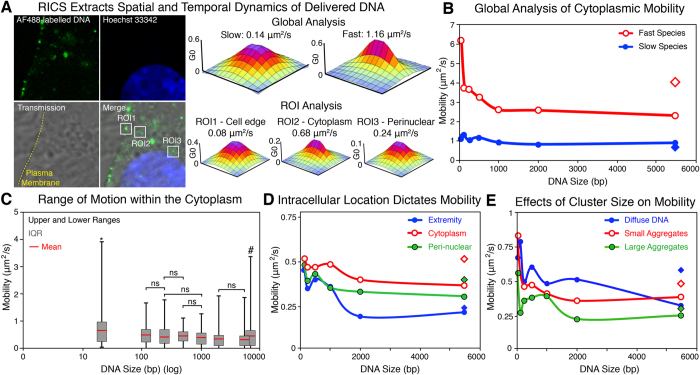
Application of RICS to Quantify Cytoplasmic Mobility. (**A**) Confocal image of myoblast cell transfected with the fluorescently labelled 495 bp fragment. RICS analysis provides quantification of mobility with 3D SCAF fits through a global analysis of the entire cytoplasm and ROI analysis of the selected ROIs along the cell edge (ROI1), cytoplasm (ROI2) and peri-nuclear region (ROI3). (Image size 12.8 × 12.8 μm). (**B**) Global analysis of delivered DNA mobility ranging from 21 bp to 5.5 kbp through RICS analysis. Two distinct species were isolated, a slow species exhibiting statistically insignificant differences (blue, filled), and a fast species (red, empty) with a size dependent decay (n = 27–51 cells). (**C**) ROI analysis of the delivered DNA demonstrates the range of motion observed within the cytoplasm of myoblasts. All DNA size exhibited complete immobility except for the 21 bp indicated by*. (# Column was shifted slightly to the right for the circular plasmid as it overlapped and covered the graph for the linear plasmid). (**D**) ROI analysis showing the influence of intracellular location on the mobility of delivered DNA along the cell extremity (blue, whole), cytoplasm (red, empty) and peri-nuclear region (green, black ring). (**E**) ROI analysis showing the effects of DNA aggregation and clustering on the delivered DNA mobility. DNA was identified as either non-clustered (diffuse) (blue, whole), small aggregates (<500 nm) (red, empty) or large aggregates (>500 nm) (green, black ring). (minimum cell number in ROI analysis = 15, minimum number of values = 83) (diamond in graphs represents circular plasmid)

Once the flDNA lipoplexes were delivered to myoblast cells, fluorescence signal was observed throughout the cell (Fig. 2A). In order to quantify the spatial temporal dynamics of the delivered flDNA two RICS methods were applied, a global analysis, which provided an insight into the mobility throughout the entire cytoplasm of the cells, and a Region-of-Interest (ROI) approach where small discrete areas (1.2 × 1.2 μm) were selected and analysed (Fig. 2A). The ROI analysis enabled specific factors influencing DNA mobility to be assessed, including the location within the cell, and cluster size of the flDNA.

The global analysis isolated two mobility species present within the cytoplasm of the cells, (i) a fast and (ii) slow species (Fig. 2A,B). The faster species exhibited a size dependent mobility, in which the smaller flDNA fragments showed significantly more rapid mobility compared to the larger fragments. On the contrary, the slower species demonstrated mobility rates that were not affected by the DNA size, and the differences were statistically insignificant (Fig. 2B and Supplementary Fig. 2A,B).

Through the RICS-ROI analysis, small regions of the cytoplasm were analysed, however only the slower species was isolated and characterised due to the fast species moving through the ROI too rapidly to be correlated. This analysis demonstrated that the flDNA across all of the DNA sizes exhibited a similar range of motion (Fig. 2C). However, the average mobility of each fragment in the cytoplasm showed a size dependent mobility across the DNA fragment sizes (Supplementary Fig. 2C). Interestingly, statistically analysis demonstrated that mobility to DNA fragments of the most proximate sizes were statistically insignificant to each other. For example, the differences in cytoplasmic mobility of the 495 bp flDNA was insignificant compared to the 240 and 1000 bp, but significantly different to all other sizes (Fig. 2C and Supplementary Fig. 2B).

The fastest rate of motion was observed in the 21 bp flDNA with an average mobility of 0.66 μm^2^/s that ranged from 0.02 to 3.92 μm^2^/s, and was the only fragment size that did not exhibit a complete immobility. Whereas, the linear plasmid exhibited the slowest cytoplasmic mobility of 0.31 μm^2^/s, ranging from 0 (immobile) to 1.89 μm^2^/s (Fig. 2C). Due to the large range of motion a relatively large variance factor was calculated, ranging from 0.37 to 0.06 (21 bp and 495 bp, respectively) (Supplementary Fig. 2A).

Through the ROI analysis, the mobility of the delivered flDNA demonstrated that the cell location dictated the rate of DNA mobility. The regions selected through this analysis were sorted to provide details on mobility along the cell margin or extremity, in the cytoplasm, and the peri-nuclear region. On average over all DNA sizes the margins of the cell exhibited the slowest mobility (0.29 μm^2^/s), which increased as the RICS analysis moved to the core of the cytoplasm (0.44 μm^2^/s). The flDNA mobility then decreased in the perinuclear region (0.36 μm^2^/s), with rates of motion in between those observed in the extremity of the cell and cytoplasm (Supplementary Fig. 3A,D). These three locations all demonstrated a size dependent mobility (Fig. 2D), however only the cell margins exhibited significant differences across the DNA fragment sizes ≥1,985 bp (Supplementary Fig. 3B). This indicates that mobility in the cytoplasm and peri-nuclear regions was not influenced by DNA size (within the DNA range assessed).

In addition to providing details on the spatial mobility of the delivered DNA, the ROI analysis also enabled the discrimination of flDNA mobility based on cluster size (Fig. 2E). Here the regions were identified as having three states, whether it was not clustered (diffuse), or as small (<500 nm diameter) or large (>500 nm diameter) clusters (Supplementary Fig. 3F). When clustered, the delivered flDNA exhibited a reduced mobility, which was further reduced as the DNA cluster size increased, as flDNA in large clusters showed a slower rate of motion than small clusters. Across the DNA sizes, the rate of motion showed a size dependent mobility in all cluster states (Fig. 2E and Supplementary Fig. 3A,C,E). It was identified that DNA maintained as clusters was associated with acidified vesicles, whereas diffuse DNA was free within the cytoplasm (Supplementary Fig. 4).

Comparing the two plasmids, which are composed of the same DNA sequence, the circular conformation had a significantly faster rate of motion compared to its linearized counterpart. The circular plasmid was on average 1.4-fold faster (0.44 μm^2^/s and 0.31 μm^2^/s, circular to linear respectively). In addition, the circular conformation displayed a maximum rate of motion 2-fold faster than the linear (3.39 μm^2^/s compared to 1.88 μm^2^/s, respectively) (Fig. 2C). Throughout the cell (i.e. cell extremity, cytoplasm and peri-nuclear), the circular plasmid exhibited a more rapid mobility to the linear form, however along the cell extremity the differences were insignificant (Fig. 2D). Conversely, once clustered the circular plasmid was consistently faster than the linear plasmid (Fig. 2E). The more rapid mobility of the circular plasmid was potentially due to supercoiling, as observed in solution (Fig. 1C).

The ROI analysis indicated that the mobility of flDNA was influenced by the location in the cell and the cluster size. Across the flDNA sizes assessed, a similar rate and range of motion was found between 120 bp to 5.5 kbp most of which were found to be statistically significant. Due to the large variance in mobility it was clear that there is no standard rate of motion in the flDNA. As a consequence, the mechanisms behind the motion were investigated through the addition of the iMSD approach.

### Determining the Mechanical Processes behind DNA Mobility

The iMSD approach was applied to further characterize the mobility of exogenous flDNA of various sizes by distinguishing between the different mechanisms that could have occurred within the cell. The iMSD analysis was able to discriminate between a variety of different mechanisms including random diffusion, active transport, subdiffusion, confined diffusion, transient confinement and binding-unbinding events (Figs 1E and Supplementary Fig. 5).

**Figure 3:**
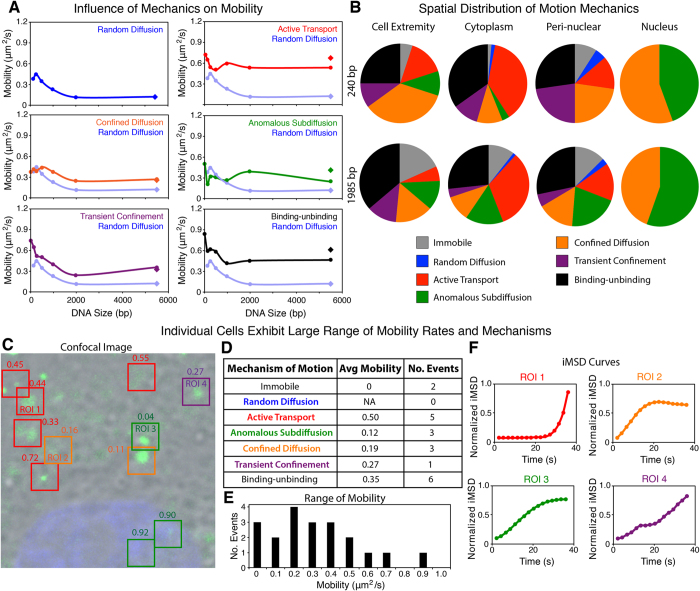
Determining the Mechanics of DNA Mobility through iMSD. (**A**) Rate of motion observed in random diffusion (blue), active transport (red), confined diffusion (orange), anomalous subdiffusion (green), transient confinement (purple) and binding-unbinding (black) events across the 21 bp–5.5 kbp DNA range within the cytoplasm of myoblasts. Random diffusion was used as a reference in the graphs comparing all other mechanisms of motion. (iMSD analysis, n = 13–17 cells) (diamonds in graphs represents circular plasmid, in random diffusion the linear and circular plasmid are the same value). (**B**) Pie charts depict the spatial distribution of each mechanism at the cell extremity, cytoplasm, peri-nuclear region and nucleus for the 240 bp and 1,985 bp DNA lipoplexes. (**C**–**F**) Individual cells exhibited a range of motion and different mechanisms within the cytoplasm. (**C**) Confocal image of a myoblast transfected with the linear plasmid lipoplex (green) and with the nucleus counterstained (blue). ROIs highlighted are coloured based on the transport mechanism present, and labelled with the rate of motion (in μm^2^/s). (Field of view 16.5 × 16.5 μm). Within the single cell a variety of mechanisms were isolated (**D**), including active transport (ROI1), confined diffusion (ROI2), subdiffusion (ROI3) and transient confinement (ROI 4) shown in iMSD graphs (**F**) and representing ROIs highlighted in image C. In iMSD curves, the iMSD y-axis was normalised to 1. (**E**) Range of motion identified within the cell shown ranging from 0–0.89 μm^2^/s.

Of the six transport mechanisms, random diffusion, confined diffusion and transient confinement exhibited a size dependent mobility. Random diffusion was the slowest transport mechanisms identified, and although confined diffusion and transient confinement were size dependent, the rate of motion observed was on average 2-fold faster than random diffusion (Fig. 3A). Transient confinement was defined as a temporary entrapment of the particle being observed, which is released during the observation.

Active transport, anomalous subdiffusion, and binding-unbinding events on the other hand demonstrated mobility rates that were independent of the DNA size (Fig. 3A). Active transport was the fastest transport mechanism observed, and in comparison to the other transport mechanisms could be identified based on the mobility rate alone (Supplementary Fig. 7). Other mechanisms including confined diffusion, anomalous subdiffusion, and transient confinement all had similar mobility rates and therefore could not be identified purely based on the rate of motion (Fig. 3A and Supplementary Fig. 7). This highlights the need for techniques such as iMSD to study the dynamics of delivered flDNA.

Interestingly, in the random diffusion, confined diffusion and transient confinement mechanisms exhibiting size dependent mobility the circular plasmids had a similar, not significantly different rate of motion to the linear plasmid. Whereas in the mechanisms not influenced by the DNA size (active transport, anomalous subdiffusion, and binding-unbinding), the circular plasmid displayed a faster rate of motion compared to the linear plasmid. It is apparent that the anomalous subdiffusion mechanism demonstrated statistically significant differences (Supplementary Fig. 6).

By investigating the mechanism of motion in the study of flDNA mobility, the variance was greatly reduced. In the ROI analysis the average variance was 0.14 ranging from 0.06–0.37 (495 bp and 21 bp, respectively) (Supplementary Fig. 2A), whereas the average variance determined from the iMSD analysis was 0.05 and ranged from 3.3^−6^−0.16 (240 bp random diffusion and 495 bp transient confinement, respectively) (Supplementary Fig. 5). This demonstrated that the specific transport mechanisms are associated to a specific rate and characteristic motion, including whether or not the mobility was affected by the flDNA size.

In addition, the iMSD approach also demonstrated a spatially distributed trend in the mechanisms of motion observed (Fig. 3B). Along the cell extremity, more non-active mechanisms were detected including confined diffusion and subdiffusion, as well as transient confinement. Binding-unbinding events typically attributed to 25–30% of events regardless of the location. Motion within the cytoplasm on the other hand was mostly attributed to active transportation. In the peri-nuclear region, the flDNA motion was mostly due to non-active mechanisms (i.e. confined diffusion and subdiffusion). Within the nucleus, only confined diffusion and subdiffusion were observed regardless of the flDNA size delivered to the cell. The only major difference between fragments was an increase in immobility as the flDNA increased in size (Fig. 3B) (charts for all flDNA sizes are shown in Supplementary Fig. 8).

The application of RICS and iMSD analysis highlighted the variation of motion of flDNA that can be observed within a single cell and population of cells. For example, the cell presented in Fig. 3C–F was transfected by a linear plasmid and displayed a variety of transport mechanisms including active transport, subdiffusion, confined diffusion, transient confinement and binding-unbinding events. This variety of mechanisms resulted in a wide range of motion from essentially immobile (0.00 μm^2^/s) to 0.9 μm^2^/s, which represents the different properties of the transport mechanisms identified. Although this analysis demonstrates that a large number of differing events can occur within a relatively small and neighbouring area, these affects are not random. As previously detailed here, specific transport mechanisms are expected to be observed in specific areas of the cell, such as, non-active mechanisms along the cell edge and peri-nuclear region, which exhibited slower mobility compared to the cytoplasm. The cytoplasm edge of the cell appears to have the slowest mobility on average. Whereas, the cytoplasm predominately contains fast, active transport and less non-active mechanisms compared to other regions of the cell cytoplasm. Examples of ROI-RICS and iMSD analysis on cells transfected with the different sized DNA fragments has been presented in Supplementary Fig. 9.

### Nuclear Mobility of flDNA

**Figure 4:**
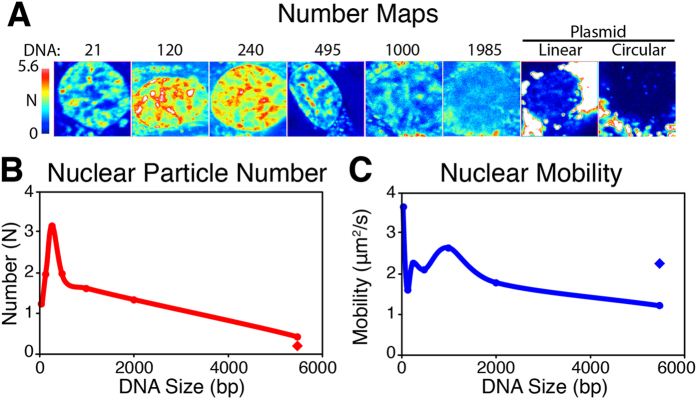
Nuclear Dynamics and Particle Number of Delivered DNA. (**A**) N&B analysis provides the number calculation with an N map showing the DNA particle number within the nucleus after 36 h. (**B**) flDNA particle number, N, and (**C**) size dependent mobility in cells transfected with a range of DNA sizes. All images are to the small scale. (n = 10 (21–1985 bp) and 8 (plasmids)) (Field of view 16.5 × 16.5 μm) (diamond in graphs represents circular plasmid).

Finally the dynamics of the flDNA within the nucleus were explored (Fig. 4). The extent of nuclear localisation was quantified through the number (N) component of the N&B approach. It was found that the 240 bp fragment accumulated in the nucleus to the greatest extent (Fig. 4A,B). The amount of flDNA detected within the nucleus decreased as the fragment size increased, where the two plasmids demonstrated the lowest amount of flDNA. The flDNA ≤1985 bp displayed a distribution throughout the nucleus, whereas the linear and circular plasmids were maintained in clusters, mostly along the edge of the nucleus.

When RICS analysis was applied to flDNA, the analysis demonstrated a rapid mobility in the nucleus compared to the cytoplasm, which fit a single species model. Within the nucleus, the flDNA had a 3.4- to 6.5-fold increase mobility compared to the cytoplasm (120 bp and 1000 bp, respectively). The overall trend in mobility displayed a size dependent decrease, however the 120–495 bp fragments were at odds with this trend (Fig. 4C).

## Discussion

Here the mobility of flDNA of various therapeutic-relevant lengths delivered through lipoplexes was quantitatively characterised using the RICS and iMSD bioimaging approaches. When used in combination, the RICS and iMSD techniques enabled non-invasive and real-time analysis of the motion of fluorescently labelled particles, in this case DNA, as well as the mechanical events attributing to the motion. Prior to this study, these imaging approaches had mostly been utilised to study endogenous protein behaviour, labelled with fluorescent proteins, such as paxillin-GFP^11^ and transmembrane transferrin receptor-GFP^12^. Therefore, this study presents alternative models for application of these FCS imaging tools, using DNA and the use of small fluorescent probes.

It has been proposed that DNA delivered through microinjection exhibits complete immobility when larger than 1000 bp^1^. Through the use of Fluorescence Recovery After Photobleaching (FRAP), Lukacs *et al.* (2000) demonstrated similar diffusive values of DNA in solution to the work presented here using RICS. The major difference appears in larger fragment sizes, in which case the findings are consistent to other reports including similar differences between the linear and circular plasmid topology^17^. Interestingly though, while Lukacs *et al.* (2000) reported complete immobility in DNA fragments greater than 1000 bp, here RICS has shown mobility in all DNA sizes addressed up to ~5.5 kbp when delivered through lipofection. Delivery approaches, more particularly the microinjection delivery^1^ may result in molecular crowding. As a consequence the cell may not process the exogenous DNA in the same manner as lipoplexes.

Through multiple particle tracking it was found that PEI-DNA complexes had a mobility ranging several magnitudes in a single cell^18^. In a similar study, a PEI-2 kbp DNA complex was found to have a similar range to our study, however the average was 2-fold slower to that reported here^19^.

Throughout this study, it was observed that the circular plasmid moved at a faster rate compared to the linear form, even though the base pair size was the same. This observation has been consistently observed in studies addressing the behaviour of DNA in different topological forms through solution DNA mobility studies^17^ and within cells^20^. The differences in DNA behaviour have been attributed to the DNA topology state, as circular plasmids have the capacity to be in relaxed or supercoiled forms, where supercoiled DNA (as in this study) demonstrate more rapid diffusion^17^ and more efficient intracellular processing^20^. Furthermore, diffusion studies have reported that linear DNA can become entangled with each other in high concentrations thereby further reducing mobility^21^.

The intracellular trafficking of delivered lipoplexes play a paramount role in the fate of the delivered DNA. Whether complexes escape from the endo-lysosome pathway may be the ultimate factor influencing the intracellular fate of delivered DNA, as failure to do so will lead to the complexes being trapped within vesicles, or lysosomal degradation^22^. The real-time trafficking of lipoplex through the endo-lysosome pathway is still poorly defined. An emphasis on polyplex delivery has highlighted that this process can be observed in real time, however no events of the DNA being released from endosomes or lysosomes was observed^23^. Time point studies however, have demonstrated that 1 h into polyplex delivery a majority of DNA is associated with endosomes. Within 4 h post transfection DNA release from endosomes into the cytoplasm was observed^24^. Furthermore, the fluorescent signal corresponding to the DNA transitioned from a clustered appearance when in endosomes, to diffuse patches throughout the cytoplasm once released^24^.

Recent work in DNA lipoplex release from endosomes indicates that these events start to occur between 2–4 h post transfection^25^. However, following escape reports propose that the free cytoplasmic DNA is rapidly degraded^25^. In the case that the delivered DNA is degraded, a change in mobility is expected to be observed, as the study presented here highlights that the motion of delivered DNA present is relative to the background event, and to the DNA size. Therefore, changes to either the trafficking event and/or DNA size are expected to alter the dynamics of the particles of interest. Additionally, in the situation that the fluorescent label is disintegrated, the DNA particle would no longer be labelled and therefore would not contribute to the analysis of these bioimaging approaches.

The biophysical mechanisms behind the delivered DNA mobility demonstrated a similar trend of a virus (influenza)^26^ and PEI-DNA complex^27^, which follows a 3 phase process of (i) slow directed into the cell, (ii) confined motion along the edge of the cell, followed by (iii) fast directed motion along microtubules. RICS and iMSD analysis of flDNA delivered to cells through lipofection demonstrate further phases of mobility including: non-active motion within the peri-nuclear area, and then confined- and sub-diffusion within the nucleus. Suh *et al.* (2004) reported that the majority of activity in the peri-nuclear region is due to confined diffusion and subdiffusion, as the DNA accumulates along the nuclear envelope. However, found 80% of PEI-DNA complexes displayed non-active motion within the entire cytoplasm^19^. Over the entire cytoplasm a 1:1:1 (active transport: binding-unbinding: other transport mechanisms) ratio was observed following RICS and iMSD analysis. These differences were attributed to the dissimilarities in the studies, including transfection reagents and cell types, but also the analysis techniques applied.

Further, the motion rate for acidified compartments of 0.45 um^2^/s^28^ and dynein-mediated transport estimated to reach up to 0.7 um^2^/s^29^, were all within the range of active transport observed within the cytoplasm of this study. The knockdown of microtubules has previously demonstrated the contribution of these structures to successful gene delivery^3^. Additionally through single particle tracking studies, and chemical microtubules knockdown a significant decrease in active transport of lipoplexes in the cytoplasm is observed^30^. Without the microtubules it has been estimated it would take 8.7 h to travel 10 μm in the cell through passive diffusion^18^.

To date, few studies have documented the dynamics of delivered DNA within the nucleus. When microinjected directly into the nucleus it has been found that DNA ranging from 21 bp to 6kbp was completely immobile^1^. However, other reports have shown DNA <400 bp injected into the cytoplasm rapidly diffused into and throughout the nucleus^31^, though the rate of motion was not determined. These two contradicting studies demonstrate that the dynamics of the DNA in the nucleus, like the cytoplasm, may rely on a number of different factors, including cell type, and DNA delivery (both studies utilised microinjection^1^,^31^).

Nevertheless, studies addressing the nuclear dynamics of other macromolecules have shown a similar range to that observed here for flDNA. Politz *et al.* (1998) found the mobility of oligonucleotides to be 9 um^2^/s^32^. Further, endogenous intranuclear mRNA, appeared to move at an average rate of 0.6 um^2^/s^33^, transcription factors ranging from 0.34–1.99 um^2^/s^34^ and nuclear proteins, HMG17, SF2/ASF and Fibrillarin diffuse at 0.45, 0.24 and 0.53 um^2^/s^35^, respectively. The mobility values of flDNA analysed via RICS and iMSD are consistent with the nuclear dynamics of other macromolecules reported above.

Within the nucleus, the delivered DNA demonstrated only two forms of motion, either confined diffusion or subdiffusion. This occurrence fits the model of nuclear dynamics of macromolecules around chromatin structures^36^. It has been reported that chromatin imposes as either, a complete barrier or a penetrable barrier where compounds are able to pass through the structure^36^. Subdiffusion could be due to the chromatin and intra-nuclear DNA and other structures acting as a barrier and crowding the space, whereas the confined diffusion, a result of either crowding within the area or as a result of the delivered DNA passing through less dense chromatin.

Within the nucleus, the delivered DNA appears to follow a size-dependent behaviour. Previous reports show that DNA ≤3,000 bp is excluded from specific nuclear compartments, including the nucleolus and Promyelocytic Leukemia (PML) bodies, but diffuse throughout the rest of the nucleus in a similar pattern to chromatin, however do not associate with the SAF-A nuclear scaffolding protein^37^. The plasmid DNA constructs (>5 kbp) on the other hand, followed a similar pattern to viral vector localisation where some DNA viral vectors move very little within the nucleus from their site of entry, resulting in accumulation of the viral vector genome along the periphery of the nucleus^38^.

The study presented here provides a novel approach to characterise gene delivery complexes. Through the application of bioimaging approaches including RICS, iMSD and N&B, it is expected that a more in depth knowledge of lipoplex delivery can be obtained. By monitoring the delivery quantitatively and in real time, it is expected that the efficiency of the approach can be enhanced by understanding key limitations and ideal reagents or approaches, and thereby make a greater impact in the clinical setting. In summary, the application of RICS and iMSD to characterise the spatial temporal dynamics of different DNA particle sizes, delivered to live cells, has enabled the quantification and the transport mechanism of these lipoplexes. The goal of this study was to assess how the DNA size influences the dynamics and mechanics of motion throughout the cell in real time. Further, this model can be applied to track various form of nucleotides, including a range of RNA species, or other macromolecules that can be fluorescently labelled. In addition, this study demonstrates the potential of the RICS and iMSD approaches for future investigations into the dynamics of delivered macromolecules.

## Materials and Methods

### Production of DNA Fragments

The 5485 bp fragment was obtained by labelling PCI-Neo mammalian vectors (Promega, Madison, WI) with Alexa Fluor 488 through a UYLSIS nucleic acid labelling kit (Molecular Probes, Eugene, OR). The labelled plasmid was either kept circular or linearized by digesting with BgI II.

Alexa Fluor488 labelled 21 bp oligonucleotide (5′-AF488-TCAATATTGGCCATTAGCCAT-3′) was synthesised (Integrated DNA Technologies, Coralville, IA) and used as a forward primer to produce 120, 240, 495, 1000 and 1985 bp fragments (reverse primers: 5′-GGACATGAGCCAATATAAATGTACA-3′, 5′-GGGCCATTTACCGTAAGTTATG-3′, 5′-ACGTAGATGTACTGCCAAGTAGGA-3′, 5′-GATGTCAGTAAGACCAATAGGTGC-3′, 5′-GTGTGAAATACCGCACAGATG-3′, respectively) using PCI-Neo plasmids as template. PCR reactions were performed using Platinum Taq DNA Polymerase (Invitrogen, Carlsbad, CA). DNA fragment lengths were confirmed through agarose electrophoresis.

The free diffusion of DNA fragments was assessed by diluting 150 ng of DNA into 250 μL of FBS-free DMEM (Invitrogen, Carlsbad, CA) as either naked DNA, or as lipoplexes (prepared as below). All solution measurements were performed at 37**°**C with the same collection setup as the cells (detailed below).

### Cell Culture and Transfection

Rat L6 myoblast cells (ATCC CRL-1458) were maintained in DMEM (Invitrogen, Carlsbad, CA) supplemented with 10% (vol/vol) FBS (Invitrogen, Carlsbad, CA). Cells were cultured at 37**°**C in a 5% CO_2_ humidified incubator in normoxia. Once suspended, 10^4^ cells were seeds into 8-well glass chamber slides (Thermo Fisher Scientific, Australia) and incubated overnight.

At 80% confluency cells were transfected using X-Treme Gene 9 (Roche, Germany), according to the manufactures’ guidelines with a 1:3 DNA:lipid ratio. To prepare lipoplexes, (at room temperature) 50 μL of serum-free DMEM was combined with 0.45 μL of lipid reagent, followed by 150 ng of fluorescently labelled DNA. The resulting mixutre was incubated for 15 min, and then added directly to cell preparations. During each experiment, 4 wells were transfected with each fragment and cells were imaged from all 4 wells. At all times cells were maintained in DMEM containing 10% FBS.

For cytoplasmic measurements, the cells were imaged between 4−8 hr after introduction of the lipoplexes, whereas, nuclear measurements were imaged after 36 hr. Cell nuclei were counterstained with Hoechst 33342 (1 μg/mL).

### Image Acquisition and Data Analysis

Confocal images and RICS, iMSD and N&B data was acquired using a Leica True Confocal Scanner – Spectro-Photometer 5 inverted confocal microscope (Leica Microsystems, Germany) equipped with a 63 × 1.4 NA water objective and 405-nm, 488-nm and 633-nm lasers for Hoechst, Alexa Fluor488 excitation and transmission images, respectively. RICS, iMSD and N&B data was acquired using Avalanche Photodiodes (APDs) fitted with a 500–550 nm filter (Leica Microsystems, Germany). The TCS-SP5 was coupled and synchronised with an ISS Vista Becker and Hickl FCS card and software (Becker & Hickl GmbH, Germany), from which the RICS, iMSD and N&B data were collected through. All RICS, iMSD and N&B data was collected with 100 frames in a 256 × 256 format with a 32 μs pixel dwell speed at 37 °C. Confocal images were collected with two channels, 415–480 nm for Hoescht 33342 and 500–550 nm for Alexa Fluor 488 labelled DNA.

RICS, N&B and iMSD data were analysed using the Globals software package, SimFCS 2.0, developed at the Laboratory for Fluorescence Dynamics at the University of California, Irvine (www.lfd.uci.edu/globals). The waist of the PSF was determined with eGFP as previously described^14^. All statistical analyses were performed with GraphPad Prism 6 software using an unpaired, two-tailed Student’s t-test, with data presented as mean ± se.

## Additional Information

**How to cite this article**: Mieruszynski, S. *et al.* Characterization of exogenous DNA mobility in live cells through fluctuation correlation spectroscopy. *Sci. Rep.* **5**, 13848; doi: 10.1038/srep13848 (2015).

## Supplementary Material

Supplementary Information
