# Supplementary material for: Characterization of exogenous DNA mobility in live cells through fluctuation correlation spectroscopy
Source: Sci Rep. 2015 Sep 10;5:13848. doi: 10.1038/srep13848 (PMC4564760; doi:10.1038/srep13848)
Supplement: Supplementary Information [file srep13848-s1.pdf]

Supplementary Information

Characterization of exogenous DNA mobility in live cells through fluctuation correlation spectroscopy

Stephen Mieruszynski [1], Michelle A. Digman [2,3], Enrico Gratton [2,3], Mark R Jones [1]*

[1] University of Western Sydney, School of Science and Health, Hawkesbury Campus, Locked Bag 1797, Penrith NSW 2751, Australia.

[2] Department of Developmental and Cell Biology, University of California Irvine, Irvine, California, United States of America; Department of Biomedical Engineering, Laboratory for Fluorescence Dynamics, University of California Irvine, Irvine, California, United States of America.

[3] *Centre for Bioactive Discovery in Health and Ageing, School of Science and Technology, University of New England, Armidale, Australia.*

** Corresponding author:*

Name: Mark R Jones

Address: University of Western Sydney, School of Science and Health, Hawkesbury Campus Building K12.53, Locked Bag 1797, Penrith NSW 2751, Australia

Telephone Number: 61 02 4570 1444

Email: m.r.jones@uws.edu.au

Supplementary Figure 1

A

	DNA Size (bp)	Mean (µm²/s)	Standard Deviation	Range (µm²/s) Minimum	Range (µm²/s) Maximum
DNA Fragment	21	55.11	4.79	45.77	64.89
	120	20.86	2.45	16.65	26.90
	240	13.16	0.81	11.46	13.92
	495	6.01	1.15	3.59	7.88
	1000	3.43	0.51	2.34	4.86
	1985	2.80	0.50	1.92	3.99
	5472 (Linear)	1.23	0.10	0.94	1.44
	5472 (Circular)	1.83	0.17	1.51	2.36
Lipoplex	21	18.65	5.58	12.95	29.76
	120	4.37	2.38	1.45	15.59
	240	2.94	0.43	2.05	3.61
	495	1.96	0.79	0.77	4.15
	1000	1.26	0.24	0.56	1.78
	1985	1.10	0.51	0.51	4.15
	5472 (Linear)	0.89	0.23	0.31	2.13
	5472 (Circular)	3.71	0.50	1.87	5.18

*All samples were separately repeated 10 times, and sampled 10 times (n = 100)

B

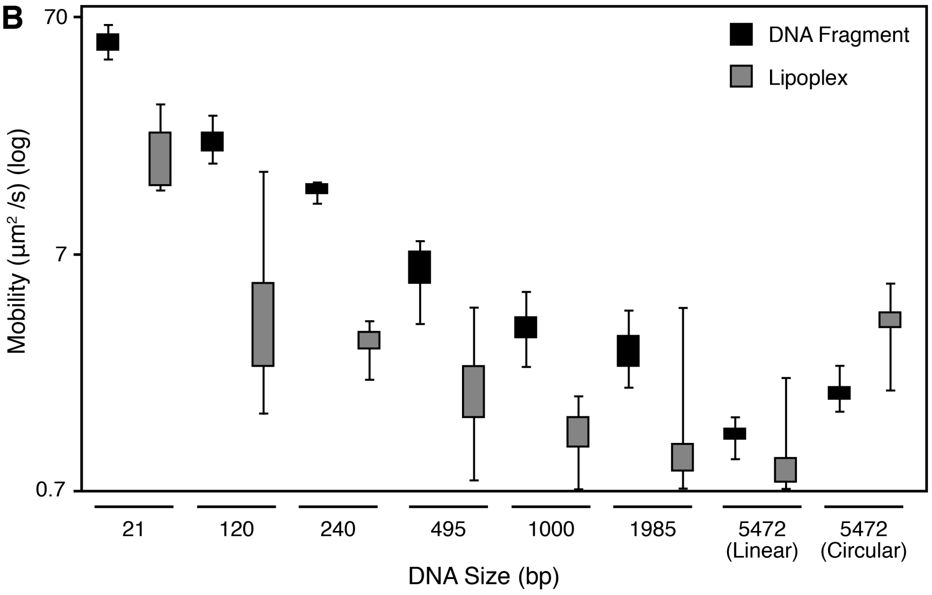

Supplementary Figure 1. RICS analysis of DNA and lipoplexes in solution. DNA of various sizes was studied either alone, or complexed as lipoplexes in solution to elucidate the intrinsic diffusion of each. (A) The values and (B) graph demonstrate a size dependent mobility when simple diffusion occurs in solution. When the DNA is linear, the lipoplexes always exhibited slower diffusion, whereas the circular plasmid had an increased rate once complexed with lipids.

Supplementary Figure 2

A

DNA Size (bp)	Slow Species from Global Analysis: Mean (µm²/s)	Standard Deviation	Variance	Range (µm²/s) Minimum	Maximum	ROI Analysis: Mean (µm²/s)	Standard Deviation	Variance	Range (µm²/s) Minimum	Maximum
21	0.97	0.65	0.42	0.20	3.05	0.66	0.61	0.37	0.02	3.92
120	1.06	1.03	1.07	0	3.80	0.48	0.34	0.12	0	1.67
240	0.83	1.17	1.38	0	4.78	0.43	0.33	0.11	0	1.79
495	0.93	1.02	1.04	0	3.64	0.44	0.25	0.06	0	1.12
1000	0.73	0.64	0.42	0	3.12	0.40	0.28	0.08	0	1.30
1985	0.65	0.65	0.43	0	2.03	0.33	0.31	0.09	0	1.94
5472 (Linear)	0.71	0.83	0.68	0	2.72	0.31	0.29	0.08	0	1.89
5472 (Circular)	0.55	0.71	0.51	0	3.42	0.44	0.45	0.20	0	3.39

B Statistical Significance between DNA Sizes

Global Analysis (Slow)

	Circular 5.5kbp	Linear 5.5kbp	1,985	1,000	495	240	120
21	*	ns	*	ns	ns	ns	ns
120	*	ns	*	ns	ns	ns	
240	ns	ns	ns	ns	ns		
495	ns	ns	ns	ns			
1,000	ns	ns	ns				
1,985	ns	ns					
Linear 5.5kbp	ns						

ROI Analysis

	Circular 5.5kbp	Linear 5.5kbp	1,985	1,000	495	240	120
21	**	****	****	**	**	**	*
120	*	****	****	*	**	ns	
240	***	***	**	ns	ns		
495	****	****	***	ns			
1,000	***	***	*				
1,985	***	ns					
Linear 5.5kbp	***						

* $p \le 0.05$
** $p \le 0.01$
*** $p \le 0.001$
**** $p \le 0.0001$
ns = no significant difference

C Size Dependent Mobility in ROI Analysis

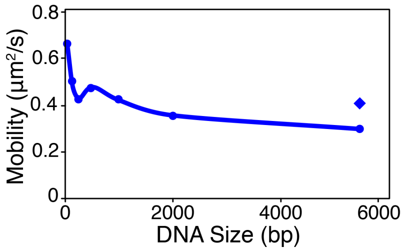

Supplementary Figure 2. Cytoplasmic mobility of delivered DNA. When transfected, the DNA was analysed through a global and ROI RICS analysis. Through the global analysis a slower species was isolated that was similar to the values obtained through the ROI analysis. (A) The mean, standard deviation, variance and upper and lower ranges of the cytoplasmic localised DNA has been presented for the slow species of the global and ROI analysis. (B) Statistical analysis was performed using a T Test assuming unequal variances showing that the global analysis resulted in mobility rates that were statistically insignificant from each other. However, in the ROI analysis, the mobility of most fragments was statistically significant. (C) The mean mobility obtained in the ROI analysis has been plotted in a scatter plot demonstrating the size dependent mobility of the DNA. Diamond in graph (C) represents the circular plasmid.

Supplementary Figure 3

A ROI data values based on cell location and DNA cluster size

	Intracellular Position			Cluster Size		
DNA Size (bp)	Cell Extremity	Cytoplasm	Peri-nuclear	Diffuse	Small Cluster	Large Cluster
21	NA	NA	NA	0.57	0.82	0.55
120	0.44	0.51	0.47	0.78	0.56	0.27
240	0.34	0.46	0.38	0.49	0.46	0.35
495	0.39	0.46	0.42	0.60	0.47	0.37
1000	0.34	0.48	0.34	0.48	0.40	0.39
1985	0.17	0.39	0.32	0.51	0.36	0.22
5472 (Linear)	0.20	0.36	0.29	0.32	0.38	0.24
5472 (Circular)	0.21	0.48	0.38	0.66	0.48	0.34

B Intraellular location significant differences

Cell Edge

	Circular 5.5kbp	Linear 5.5kbp	1,985	1,000	495	240
120	***	***	***	ns	ns	ns
240	*	**	**	ns	ns	
495	***	***	****	ns		
1,000	**	**	***			
1,985	ns	ns				
Linear 5.5kbp	ns					

Cytoplasm

	Circular 5.5kbp	Linear 5.5kbp	1,985	1,000	495	240
120	ns	***	***	ns	ns	ns
240	ns	**	ns	ns	ns	
495	ns	**	ns	ns		
1,000	ns	***	ns			
1,985	ns	ns				
Linear 5.5kbp	**					

Peri-nuclear

	Circular 5.5kbp	Linear 5.5kbp	1,985	1,000	495	240
120	ns	**	**	ns	ns	ns
240	ns	ns	ns	ns	ns	
495	ns	ns	ns	ns		
1,000	ns	ns	ns			
1,985	ns	ns				
Linear 5.5kbp	**					

C DNA cluster size significant differences

Diffuse

	Circular 5.5kbp	Linear 5.5kbp	1,985	1,000	495	240	120
21	ns	**	*	**	*	***	**
120	ns	****	***	****	**	***	
240	ns	**	ns	ns	ns		
495	ns	***	ns	ns			
1,000	***	**	ns				
1,985	ns	**					
Linear 5.5kbp	***						

Small Clusters

	Circular 5.5kbp	Linear 5.5kbp	1,985	1,000	495	240	120
21	**	***	***	***	**	**	*
120	ns	***	***	***	ns	ns	
240	ns	**	*	ns	ns		
495	ns	ns	***	ns			
1,000	ns	*	ns				
1,985	**	ns					
Linear 5.5kbp	****						

Large Clusters

	Circular 5.5kbp	Linear 5.5kbp	1,985	1,000	495	240	120
21	**	***	****	*	**	**	***
120	ns	ns	ns	**	**	ns	
240	ns	**	**	ns	ns		
495	ns	***	***	ns			
1,000	ns	***	***				
1,985	***	ns					
Linear 5.5kbp	**						

D Mobility in cell locations across all DNA sizes

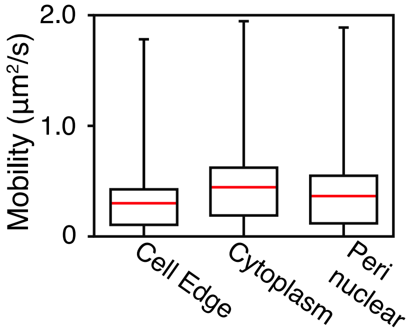

E Mobility of DNA cluster sizes across all DNA sizes

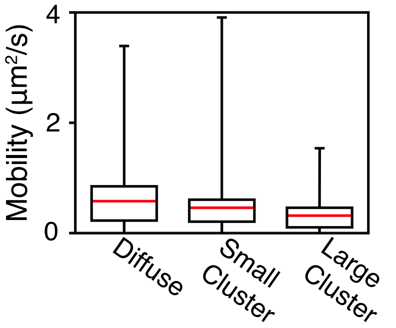

F Example of DNA cluster sizes

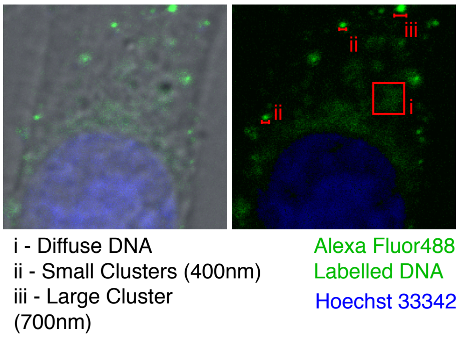

Supplementary Figure 3. Influence of intracellular location and DNA clustering on DNA mobility. Through the ROI analysis within the cytoplasm, data was sorted to demonstrate the

influence of the cell position, and cluster size on mobility. (A) The values obtained have been presented, demonstrating a more rapid mobility within the cytoplasm, compared to the cell extremity and peri-nuclear regions. When the DNA did not cluster, it had demonstrated a faster rate of motion. In many cases a range of cluster sizes were observed within a single cell, including DNA that had not clustered. (B) Statistical differences determined between DNA sizes at the cell edge, cytoplasm and peri-nuclear regions. (C) Statistical differences between DNA sizes when DNA is contained within the cytoplasm as diffuse DNA, small clusters and large clusters. (ns = no significantly difference, * $p \leq 0.05$, ** $p \leq 0.01$, *** $p \leq 0.001$, **** $p < 0.0001$). (D) Mobility throughout cell at the cell edge, cytoplasm and perinuclear regions with all DNA sizes combined. Cytoplasm exhibited the fastest mobility, and the cell edge the slowest. (E) Mobility in different cluster forms (diffuse, small and large) all DNA sizes combined. Diffuse DNA exhibited the fastest mobility, and large clusters the slowest. (F) Example of transfected cell with different DNA cluster sizes and diffuse DNA. Image size 12.8x12.8 μm.

Supplementary Material and Methods

LysoTracker and Membrane Staining

LysoTracker Red DND-99 (Molecular Probes, OR, USA) was diluted in FBS-free DMEM to a final concentration of 60 nM. Laurdan (6-dodecanoyl-2-dimethylamino naphthalene; Molecular Probes, OR, USA) was dissolved in dimethylsulfoxide (DMSO) and prepared at a 1.8 mM stock solution. Laurdan was diluted 1:1000 into FBS-free media containing diluted LysoTracker Red. Media containing dyes was prepared as required and kept at 37°C prior to addition to cells.

Cells were transfected with AF488 labelled PCI-Neo plasmid (circular) as outlined in main article. At 6h post addition of DNA lipoplexes, media was aspirated and media containing dyes added. Cells were incubated with dyes for 1h at 37°C, and then washed twice in DMEM (containing 10% FBS v/v). Cells were imaged immediately.

Confocal Microscopy

Confocal images were obtained using a Leica TCS-SP5 equipped with a 63X 1.4 NA water objective. To image Laurdan, AF-488 labelled DNA, and LysoTracker Red images 405 nm, 488 nm, and 568nm laser lines were activated acquiring 415 – 480 nm, 500 – 550 nm and 580 – 630 nm bandwidths, respectively.

Supplementary Figure 4

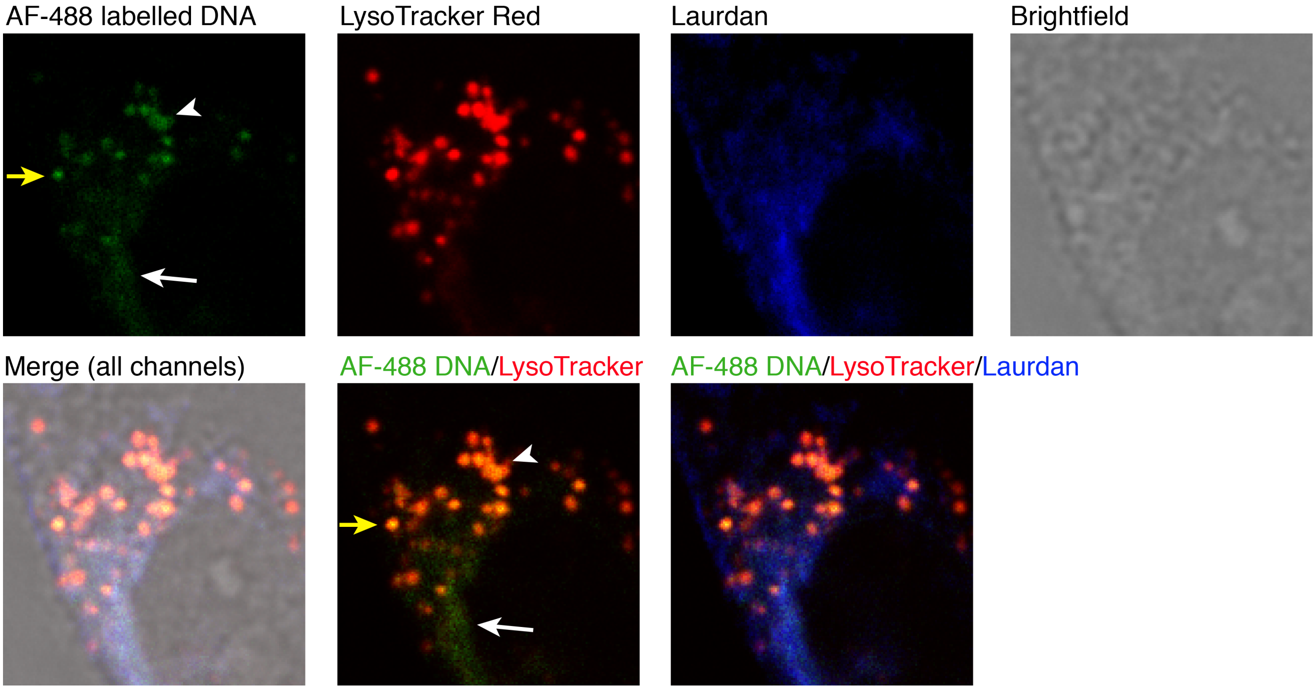

Supplementary Figure 4. Delivered DNA clusters colocalize with acidified compartments. Myoblast cell transfected with Af-488 labelled circular PCI-Neo plasmid (green), counterstained for acidified compartments (Red, LysoTracker Red) and cell membranes (blue, Laurdan) with brightfield. Small clusters (yellow arrow) and large clusters (white arrow head) colocalise with acidified compartments, whereas diffuse DNA (white arrow) does not. Image size 12.8x12.8 µm.

Supplementary Figure 5

A Random Diffusion

DNA Size (bp)	Mean (μm^2/s)	Standard Deviation	Variance	Range (μm^2/s)	
				Minimum	Maximum
21*	NA				
120	0.40	0.39	0.16	0.09	1.02
240	0.45	0.002	3.3^{-6}	0.44	0.46
495	0.34	0.12	0.04	0.09	0.44
1000	0.22	0.18	0.03	0.03	0.46
1985	0.12	0.004	1.5^{-5}	0.11	0.13
5472 (Linear)	0.13	0.06	0.01	0.08	0.15
5472 (Circular)	0.13	0.03	0.001	0.10	0.15

*No events isolated

B Active Transport

DNA Size (bp)	Mean (μm^2/s)	Standard Deviation	Variance	Range (μm^2/s)	
				Minimum	Maximum
21	0.71	0.44	0.14	0.16	1.89
120	0.64	0.35	0.12	0.12	1.67
240	0.54	0.39	0.16	0.03	1.78
495	0.50	0.21	0.04	0.15	1.05
1000	0.59	0.27	0.08	0.13	1.29
1985	0.53	0.31	0.09	0.10	1.66
5472 (Linear)	0.53	0.23	0.05	0.12	0.88
5472 (Circular)	0.67	0.33	0.11	0.21	1.49

C Anomalous Subdiffusion

DNA Size (bp)	Mean (μm^2/s)	Standard Deviation	Variance	Range (μm^2/s)	
				Minimum	Maximum
21	0.51	0.14	0.02	0.31	0.68
120	0.21	0.14	0.02	0.05	0.52
240	0.34	0.18	0.03	0.12	0.52
495	0.32	0.19	0.04	0.09	0.75
1000	0.28	0.13	0.02	0.06	0.54
1985	0.38	0.25	0.06	0.10	0.96
5472 (Linear)	0.26	0.21	0.04	0.01	0.89
5472 (Circular)	0.42	0.18	0.03	0.16	0.77

D Confined Diffusion

DNA Size (bp)	Mean (μm^2/s)	Standard Deviation	Variance	Range (μm^2/s)	
				Minimum	Maximum
21	0.38	0.29	0.089	0.02	1.39
120	0.43	0.34	0.11	0.12	1.14
240	0.40	0.26	0.07	0.03	1.09
495	0.45	0.20	0.04	0.10	0.90
1000	0.37	0.25	0.07	0.06	0.81
1985	0.24	0.18	0.03	0.04	0.65
5472 (Linear)	0.26	0.19	0.04	0.04	0.74
5472 (Circular)	0.26	0.22	0.05	0.09	0.67

E Transient Confinement

DNA Size (bp)	Mean (μm^2/s)	Standard Deviation	Variance	Range (μm^2/s)	
				Minimum	Maximum
21	0.73	0.21	0.15	0.09	1.65
120	0.63	0.23	0.05	0.08	0.88
240	0.52	0.29	0.09	0.09	1.24
495	0.49	0.35	0.16	0.11	1.12
1000	0.39	0.37	0.13	0.02	1.39
1985	0.24	0.15	0.02	0.01	0.48
5472 (Linear)	0.35	0.17	0.03	0.14	0.55
5472 (Circular)	0.33	0.22	0.05	0.10	0.67

F Binding-unbinding

DNA Size (bp)	Mean (μm^2/s)	Standard Deviation	Variance	Range (μm^2/s)	
				Minimum	Maximum
21	0.74	0.37	0.14	0.13	1.28
120	0.46	0.33	0.11	0.03	1.39
240	0.49	0.36	0.12	0.05	1.78
495	0.46	0.24	0.06	0.05	1.06
1000	0.32	0.29	0.08	0.05	0.97
1985	0.37	0.34	0.12	0.03	1.94
5472 (Linear)	0.36	0.34	0.11	0.03	1.88
5472 (Circular)	0.49	0.39	0.15	0.01	2.05

Supplementary Figure 5. Relationship between transport mechanisms and motion within the cytoplasm. The mean, standard deviation, variance and upper and lower range values for the different mechanisms of motion including random diffusion (A), active transport (B), anomalous subdiffusion (C), confined diffusion (D), transient confinement (E) and binding-unbinding (F).

Supplementary Figure 6

Significant differences in iMSD analysis across DNA sizes

A Random Diffusion

	Circular 5.5kbp	Linear 5.5kbp	1,985	1,000	495	240	120
21	na	na	na	na	na	na	na
120	**	**	**	*	ns	ns	
240	****	****	****	****	****		
495	****	****	****	***			
1,000	**	**	**				
1,985	ns	ns					
Linear 5.5kbp	ns						

B Active Transport

	Circular 5.5kbp	Linear 5.5kbp	1,985	1,000	495	240	120
21	ns	**	**	ns	**	**	ns
120	ns	ns	ns	ns	ns	ns	
240	ns	ns	ns	ns	ns		
495	ns	ns	ns	ns			
1,000	ns	ns	ns				
1,985	ns	ns					
Linear 5.5kbp	ns						

C Confined Diffusion

	Circular 5.5kbp	Linear 5.5kbp	1,985	1,000	495	240	120
21	**	*	**	*	ns	ns	ns
120	**	**	*	ns	ns	ns	
240	*	*	*	ns	ns		
495	*	*	**	ns			
1,000	ns	ns	**				
1,985	ns	ns					
Linear 5.5kbp	ns						

D Anomalous Subdiffusion

	Circular 5.5kbp	Linear 5.5kbp	1,985	1,000	495	240	120
21	ns	**	ns	**	**	ns	***
120	**	ns	**	ns	ns	ns	
240	ns	ns	ns	ns	ns		
495	ns	ns	ns	ns			
1,000	ns	ns	ns				
1,985	ns	ns					
Linear 5.5kbp	**						

E Transient Confinement

	Circular 5.5kbp	Linear 5.5kbp	1,985	1,000	495	240	120
21	**	**	***	**	*	ns	ns
120	**	**	****	**	ns	ns	
240	**	***	****	ns	ns		
495	*	**	***	ns			
1,000	ns	ns	**				
1,985	*	**					
Linear 5.5kbp	ns						

F Binding-unbinding

	Circular 5.5kbp	Linear 5.5kbp	1,985	1,000	495	240	120
21	ns	ns	ns	ns	ns	ns	ns
120	ns	ns	ns	ns	ns	ns	
240	ns	ns	ns	ns	ns		
495	ns	ns	ns	ns			
1,000	ns	ns	ns				
1,985	ns	ns					
Linear 5.5kbp	ns						

* $p \leq 0.05$
** $p \leq 0.01$
*** $p \leq 0.001$
**** $p \leq 0.0001$
ns = no significant difference

Supplementary Figure 6. Significant differences in transport mechanisms across DNA sizes.

Supplementary Figure 7

Significant differences in iMSD analysis between transport mechanisms

A 21 bp

	B-UB	TC	CD	AS	AT
RD	na	na	na	na	na
AT	*	ns	***	**	
AS	ns	ns	ns		
CD	ns	**			
TC	ns				

B 120 bp

	B-UB	TC	CD	AS	AT
RD	**	***	ns	***	**
AT	**	***	*	****	
AS	***	****	*		
CD	ns	ns			
TC	ns				

C 240 bp

	B-UB	TC	CD	AS	AT
RD	ns	ns	**	ns	***
AT	ns	ns	**	***	
AS	**	**	ns		
CD	**	***			
TC	ns				

D 495 bp

	B-UB	TC	CD	AS	AT
RD	***	**	ns	ns	****
AT	ns	ns	**	**	
AS	*	*	ns		
CD	**	*			
TC	ns				

E 1,000 bp

	B-UB	TC	CD	AS	AT
RD	***	**	**	ns	****
AT	***	***	***	****	
AS	**	ns	ns		
CD	ns	ns			
TC	ns				

F 1,985 bp

	B-UB	TC	CD	AS	AT
RD	****	****	***	****	****
AT	**	****	****	**	
AS	ns	**	**		
CD	*	ns			
TC	**				

G Linear 5.5 kbp plasmid

	B-UB	TC	CD	AS	AT
RD	****	****	***	***	****
AT	**	***	***	****	
AS	ns	ns	ns		
CD	ns	ns			
TC	ns				

H Circular 5.5 kbp plasmid

	B-UB	TC	CD	AS	AT
RD	****	***	***	***	****
AT	**	****	****	***	
AS	ns	ns	**		
CD	***	ns			
TC	**				

* p ≤ 0.05
** p ≤ 0.01
*** p ≤ 0.001
**** p ≤ 0.0001
ns = no significant difference

Supplementary Figure 7. Significant differences in between transport mechanisms.

Supplementary Figure 8

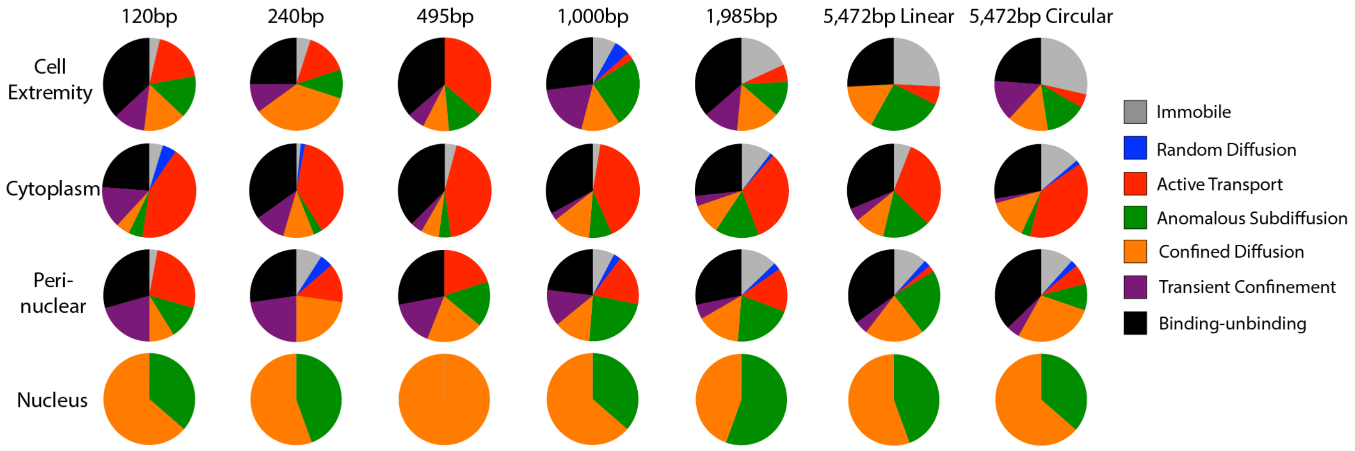

Supplementary Figure 8. Spatial distribution of transport mechanisms. The iMSD analysis demonstrated a preference of certain transport mechanisms depending of the location of the cell. This trend was independent of the size of DNA present, as the only significant changes in transport mechanisms was an increase in immobility as the DNA length increased. This figure shows the transport mechanisms for the range of DNA sizes assessed.

Supplementary Figure 9

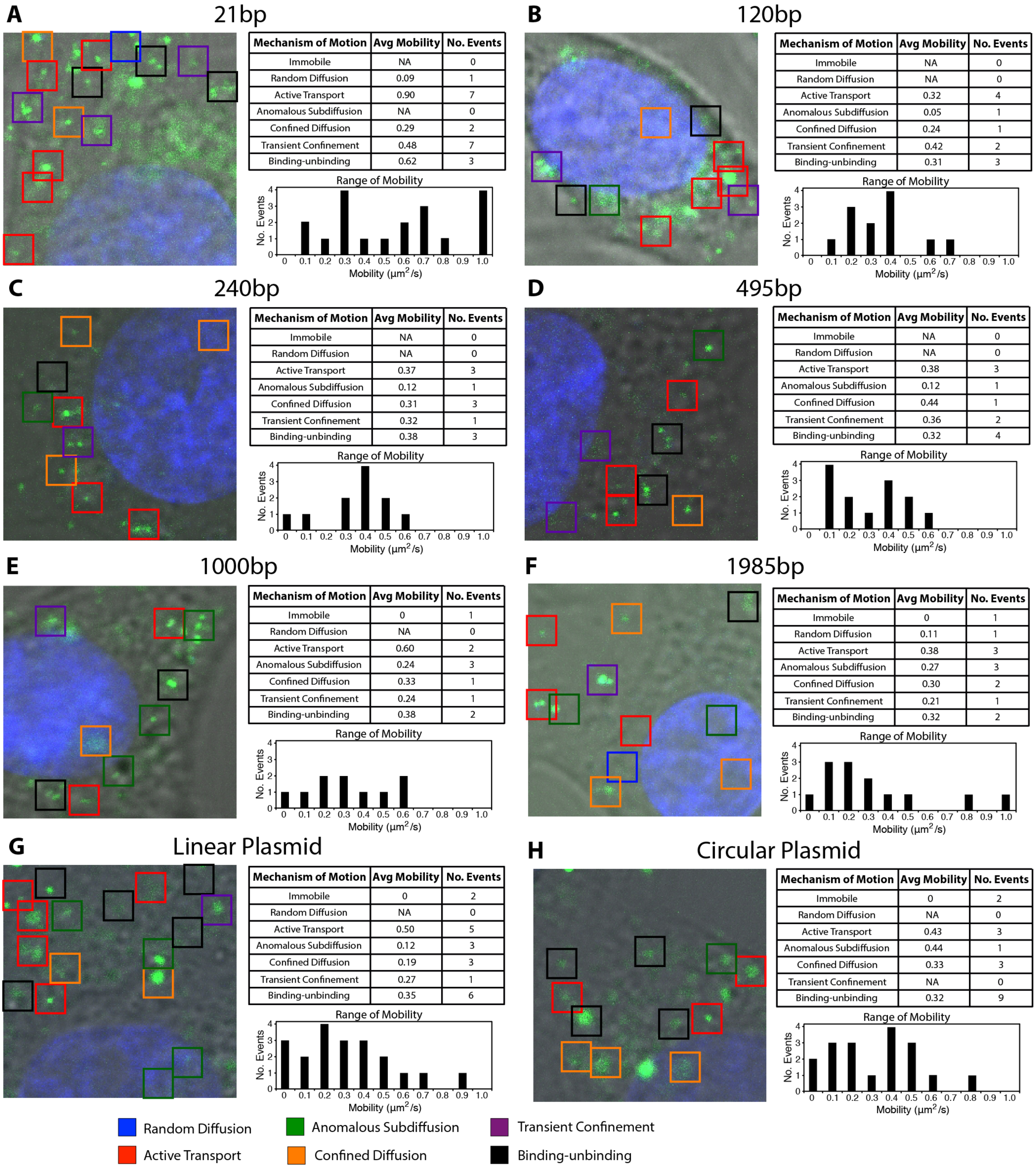

A – 21bp

Mechanism of Motion	Avg Mobility	No. Events
Immobile	NA	0
Random Diffusion	0.09	1
Active Transport	0.90	7
Anomalous Subdiffusion	NA	0
Confined Diffusion	0.29	2
Transient Confinement	0.48	7
Binding-unbinding	0.62	3

B – 120bp

Mechanism of Motion	Avg Mobility	No. Events
Immobile	NA	0
Random Diffusion	NA	0
Active Transport	0.32	4
Anomalous Subdiffusion	0.05	1
Confined Diffusion	0.24	1
Transient Confinement	0.42	2
Binding-unbinding	0.31	3

C – 240bp

Mechanism of Motion	Avg Mobility	No. Events
Immobile	NA	0
Random Diffusion	NA	0
Active Transport	0.37	3
Anomalous Subdiffusion	0.12	1
Confined Diffusion	0.31	3
Transient Confinement	0.32	1
Binding-unbinding	0.38	3

D – 495bp

Mechanism of Motion	Avg Mobility	No. Events
Immobile	NA	0
Random Diffusion	NA	0
Active Transport	0.38	3
Anomalous Subdiffusion	0.12	1
Confined Diffusion	0.44	1
Transient Confinement	0.36	2
Binding-unbinding	0.32	4

E – 1000bp

Mechanism of Motion	Avg Mobility	No. Events
Immobile	0	1
Random Diffusion	NA	0
Active Transport	0.60	2
Anomalous Subdiffusion	0.24	3
Confined Diffusion	0.33	1
Transient Confinement	0.24	1
Binding-unbinding	0.38	2

F – 1985bp

Mechanism of Motion	Avg Mobility	No. Events
Immobile	0	1
Random Diffusion	0.11	1
Active Transport	0.38	3
Anomalous Subdiffusion	0.27	3
Confined Diffusion	0.30	2
Transient Confinement	0.21	1
Binding-unbinding	0.32	2

G – Linear Plasmid

Mechanism of Motion	Avg Mobility	No. Events
Immobile	0	2
Random Diffusion	NA	0
Active Transport	0.50	5
Anomalous Subdiffusion	0.12	3
Confined Diffusion	0.19	3
Transient Confinement	0.27	1
Binding-unbinding	0.35	6

H – Circular Plasmid

Mechanism of Motion	Avg Mobility	No. Events
Immobile	0	2
Random Diffusion	NA	0
Active Transport	0.43	3
Anomalous Subdiffusion	0.44	1
Confined Diffusion	0.33	3
Transient Confinement	NA	0
Binding-unbinding	0.32	9

Supplementary Figure 9. Examples of cells transfected with DNA of various sizes. An example for each DNA fragment have been presented including 21bp (A), 120bp (B), 240bp (C), 495bp (D), 1000bp (E), 1985bp (F) and, linear (G) and circular (H) plasmid has been presented. Each example has ROIs selected representative of the different mechanisms of motion identified in each cell (legend at the bottom of the figure). In addition summaries of the types of mechanisms found and range of motion observed have been presented.
